# From PBL tutoring to PBL coaching in undergraduate medical education: an interpretative phenomenological analysis study

**DOI:** 10.3402/meo.v21.31973

**Published:** 2016-07-08

**Authors:** Qing Wang, Huiping Li, Weiguo Pang

**Affiliations:** 1 School of Psychology and Cognitive Science, East China Normal University, Shanghai, China; 2 Department of Respiratory Medicine, Shanghai Pulmonary Hospital, School of Medicine, Tongji University, Shanghai, China

**Keywords:** problem-based learning, coaching, coaching psychology, medical education, IPA

## Abstract

**Background:**

Coaching psychology is of increasing interest to medical educators for its potential benefits as a facilitative method in problem-based learning (PBL). However, the field lacks empirical studies that explore the lived experiences of students and tutors in the PBL coaching process. This study aimed to elicit knowledge regarding medical students’ and tutors’ experiences and perceptions of PBL coaching in the context of Chinese undergraduate medical education.

**Methods:**

The qualitative methodology of interpretative phenomenological analysis (IPA) was employed. Participants comprised third year medical students (*n=*20) and PBL tutors (*n=*5) who have adopted a coaching approach in PBL for a semester. Semi-structured interviews were utilized to obtain a comprehensive understanding of their experiences of PBL coaching. Data analysis followed an iterative four-stage scheme of Biggerstaff and Thompson.

**Results:**

Six main themes emerged from diverse experiences and interpretations: 1) mindsets of coaching and learning, 2) the development of learning dispositions and capacities, 3) student group collaboration, 4) tutor–student relationships, 5) personal and professional development, and 6) challenges and difficulties in implementation.

**Conclusions:**

It could be concluded that PBL coaching is a dynamic, facilitative process that makes a particular contribution to the learning process from psychological, emotional, and social perspectives, whilst it demonstrates significant overlaps with PBL tutoring in terms of supporting students’ cognitive activities in PBL. Further research is needed to identify the barriers and challenges for medical educators to implement coaching in the PBL process.

Problem-based learning (PBL) is a learner-centered instructional method by which students learn content knowledge, thinking strategies, and self-directed learning skills through experiential learning and facilitated, collaborative problem-solving (1, 2). In medical education, PBL provides students with guided experience in the context of complex, authentic patient problems and allows the students to take responsibility for their own learning and develop intrinsic motivation, flexible knowledge, clinical logicality, and effective self-directed and collaborative learning skills (3, 4).

PBL tutoring, as a facilitative method, is of critical importance to the effective functioning of PBL (5). It primarily requires an understanding of the learning process to create a supportive learning environment that encourages active participation by all members and continuously monitors the quality of learning. The literature varies widely on the characteristics of effective PBL tutors, especially on tutor's role as a content expert or/and a process expert. For example, Barrows and Tamblyn believed that ‘the tutor should have expertise in group facilitation (process expertise) rather than in a subject area (content expertise)’ (6). Meanwhile, other studies showed that most effective tutors are those with both clinical content knowledge and the abilities to facilitate learning process and to empathize with students’ circumstances (7–9). We acknowledge that the sophisticated role of PBL tutor indicates striking the right balance between process facilitation and information delivery, a balance that alters at different points in the PBL process (3, 10). PBL tutors enable students to become increasingly competent through modeling, scaffolding, mentoring, structuring tasks and hinting without explicitly giving final answers, and progressively retreating as students become more experienced with PBL yet continuing to monitor the group and make moment-to-moment decisions on the best time to offer support (11). In general, PBL facilitation is a subtle and complicated process in which new skills must be acquired and multiple roles need to be redefined as the PBL tutors make the transition from a more traditional lecturing role to a multi-faceted role as mentor, coach, model, and guide (6, 8, 12, 13).

We believe that the extensive studies on coaching and coaching psychology have invigorated the domain of PBL facilitation. Coaching can be seen as a process of human development that involves focused interaction between the coaches and the coachees, and the use of appropriate strategies, tools, and techniques in order to promote desirable and sustained cognitive, emotional, and behavioral changes that facilitate well-being, goal attainment, and enhanced performance in work and personal life domains with non-clinical populations (14, 15). The purpose of coaching is to unlock people's potential to maximize their performance and help them to learn rather than teaching them (16). Coaching, in order to be theory-informed and evidence-based, should be grounded in established learning or psychological approaches (17). The most widely used psychological approaches in coaching include but not limited to humanistic and person-centered approaches (18, 19), cognitive behavioral approaches (20, 21), narrative approaches (22, 23), Gestalt approaches (24, 25), and positive psychology approaches (26, 27).

In the field of education, coaching is regarded as a learning methodology with boundaries that are sometimes blurred (28). Learning is central to effective coaching: coaches facilitate the learning process; coaches, as well as the people they coach, are learners (29). Theoretically, coaching is particularly related to adult learning that emphasizes the role of prior experience and self-directed learning (30, 31), experiential learning that stresses the role of current experience and reflection (32), situated learning and the interpersonal context (33), social learning and the extension of the zone of proximal development (34), and transformative learning and critical reflection (35). In practice, coaching for learning is particularly concerned with how coaches and learners establish and maintain a dialogical and participatory relationship through effective communication, so that they construct knowledge, develop lifelong learning capacities, and foster self-determination and ownership of learning through experiential, situated, and authentic learning contexts and activities. The role of coaches emphasizes intellectual and affective involvement, careful listening, acceptance, empathy, and reflection to create a non-threatening and non-judgmental environment in which learners feel free to inquire into their own experiences and seek solutions to their own problems (36).

‘Coaching’ has been mentioned by a few researchers in facilitating cognitive development, such as critical thinking or clinical logicality in the PBL process (6, 8, 11–13). Nonetheless, we argue that coaching may not be intrinsic to PBL facilitation, and it has been overlooked particularly in the context of Chinese medical education (37–39). A clear distinction between PBL tutoring and PBL coaching could be made in the following aspects. 1) PBL coaching considers personal qualities of coaches from a humanistic perspective, stressing coaches’ authenticity, and unconditional positive regards toward learners. 2) PBL coaching explicitly considers empathy and medical humanity as one of the learning goals in medical education through modeling empathetic behaviors and displaying effective communication in a non-didactic way. 3) PBL coaching pays particular attention to the emotional and motivational aspects of learners, so that emotional scaffolding, which involves establishing rapport and trust and maintaining a nurturing relationship with students, is regarded as essential, in addition to cognitive scaffolding. 4) Both PBL tutoring and PBL coaching involve drawing from students’ own resources and encouraging them to come up with their own solutions, though PBL coaching emphasizes the flexible use of various coaching skills such as active listening, questioning, probing, summarizing, reflecting, and dealing with learners’ emotions. There is scarcely any empirical investigation into PBL coaching in medical education. A recent participatory research has developed an integrated model of coaching psychology and PBL, aiming to develop students’ lifelong learning dispositions, empathetic skills, and personal psychological well-being to assist them throughout their medical careers in the future (40). The authors suggested that these ‘soft’ qualities and capacities cannot be taught in a didactic teaching method or through rigidly structured training. Rather, they might be intentionally and dexterously cultivated by particular facilitative methods such as coaching. The study explicitly combined the essential features of coaching and PBL. However, although the model was generated by medical students and tutors addressing their real educational needs, it has not been applied to a real-world PBL environment and represents a PBL variant rather than a facilitative model. The question remains about exactly how medical students and tutors perceive the PBL coaching process.

An empirical study on PBL coaching is timely because the rise of coaching and coaching psychology in the field of education may indicate a new perspective of PBL facilitation. Nonetheless, how it actually facilitates students’ learning is still unclear to many medical education researchers and practitioners. Therefore, as the significant first step, exploring students’ and tutors’ experiences of PBL coaching in real medical education settings would be necessary.

The principal research question is, ‘what are students' and tutors' experiences and perspectives of PBL coaching in the context of undergraduate medical education?’ We would employ the methodology of interpretative phenomenological analysis (IPA) to investigate this question. In this paper, we adopt the term ‘tutors’ rather than ‘coaches’ to describe those who facilitate students’ PBL, for the sake of consistency and clarity.

## Methods

### Research design

IPA is a qualitative research methodology specifically concerned with a detailed examination of the realistic account of individual's lived experience, and how a person currently constructs and uniquely makes sense of that experience using careful and systematic procedures. IPA is not interested in generalizing to a population but in relating to the specifics of the individual's constructed world. In this sense, IPA is modest in terms of its scope. The approach depends heavily on a phenomenological stance that views a person's own perception of the world as primary, holding that the focus of psychological life is the immediate consciousness (41). However, IPA is not just a description of participants’ experiences presented in the interview transcripts. It acknowledges the engagement and the reflexive role of researchers in the interpretation of the participants’ texts (42, 43). This notion of interpretation arises from hermeneutics that emphasize the way in which we interact with the texts (44). In this study, we were aware that the appropriate perspective of analysis did not come automatically. One must constantly consult the texts and other possible theoretical approaches in order to arrive at an explicit awareness of the phenomenon to be studied and settle on an appropriate angle (45). We attempted to make the analysis itself a dialectical process, in which we conceptualized and systematically presented what the participants had said, and we articulated and clarified the meanings that emerged from the data.

### Research context

The research was conducted at the Medical School of Tongji University in China in the academic year 2014–2015. At this medical school, a systematic program has been developed integrating problem-, lecture-, clinical-, and competence-based learning. The duration of the undergraduate medical education program is 5 years, which leads to a bachelor's degree (commonly called a ‘5-year program’), and the combined program is 7 years, which leads to a master's degree (commonly called the ‘7-year program’). In these two programs, the curriculum in the first 4 years are exactly the same, consisting of discipline-based modules, such as basic science, biochemistry, immunology, molecules, pharmacology, genetics, respiratory, Chinese medicine, and so on. Each year consists of four modules that last 8 weeks each. Modules are structured on the basis of lectures, PBL sessions, laboratory practices, and evidence-based medicine. PBL is included primarily in the second and third year, and each module includes two PBL scenarios. At the beginning of the second year, students are introduced with the concept, the process, the roles of learners and tutors, and the assessment process of PBL. The PBL tutors, mainly from clinical science faculty, attend a week's PBL tutoring course prior to the sessions.

In this study, the integrated framework of coaching psychology and PBL (40) was adopted in the medical school because the teaching faculty was interested in incorporating coaching into their existing PBL approach. The faculty launched a 4-day course on coaching psychology that covered the philosophy, the process, the role of coaches and learners, and essential skills of coaching, delivered by an accredited coaching psychologist who holds a PhD in the field. In order to experiment with PBL coaching on a small scale, the faculty decided to incorporate coaching only in the module of respiratory in the academic year 2014–2015. One hundred undergraduate students from the 5-year program (68%) and the 7-year program (32%) took the module. All the students were in their third year, majoring in clinical medicine. They have taken various PBL modules in their second year, with the exception of only one student, an exchange international student from Pakistan who had never taken a PBL course. Eight students have attended the course on coaching psychology. For most students, it was the first time that they would be instructed in a coaching approach. Six respiratory doctors functioned as tutors in the PBL session. Two of them were professionally trained as PBL facilitators in the US, one in Taiwan, and the other three were domestically trained in the Medical School of Tongji University. All of them had at least 2 years’ experience with PBL tutoring. All of them have taken the coaching training course and acquired varying levels of coaching experience and skills.

In the current PBL sessions, 15–18 students worked together with a tutor, and there were six parallel groups. There were two PBL scenarios, each was processed in three sessions of 2 h each over 3 weeks. During the sessions, tutors were consciously interacting with students in a coaching approach and students were expected to work collaboratively on the scenarios. Between the sessions, students were expected to study independently and interdependently toward their learning objectives as defined in the previous session. They reported an average of 5 h of self-directed learning and 3 h collaborative learning per week throughout the sessions.

### Participants

**Fig. 1:**
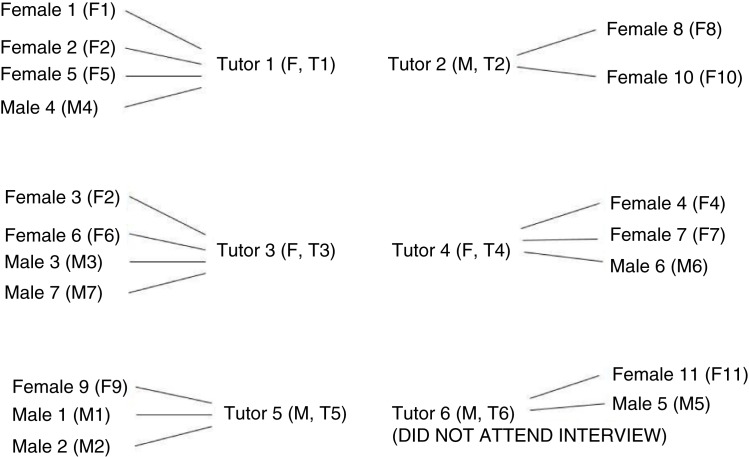
Student and tutor participants in the IPA study.

The students and tutors were informed about the IPA study and invited to participate in the interviews. Twenty students (mean of age=20.9, F=11, M=9) and five tutors (mean of age=37.2, F=3, M=2) voluntarily joined in the study. They were numbered in the order that they signed up for the interviews. Only one tutor declined the interview invitation due to urgent clinical work. Among the student participants, two female students and one male student have taken the coaching training course. All tutor participants have taken the coaching training course. The students were from different PBL groups coached by different tutors and their relationships were described in Fig. 1.

### Data collection

Prior to the formal interviews, we conducted pilot interviews and developed a ‘prompt sheet’ with a few main points for discussion. For example, ‘How do you perceive yourself as a learner/tutor in PBL coaching?’, ‘What do you think of PBL coaching?’, ‘Is PBL coaching different from the PBL tutoring that you have experienced before? If so, what are the main differences?’, ‘How does PBL coaching impact on your learning?’, ‘What issues that you would like to raise about the current PBL sessions?’ The sheet was merely the basis for conversation; it was not intended to be prescriptive and certainly not limiting in the sense of overriding the expressed interests of the participants. Twenty-five interviews were conducted in Chinese, each lasting from 30 to 55 min (with an average length of 45 min). All the interviews were audio-recorded, then transcribed into Chinese texts, and translated into English with meticulous accuracy.

### Data analysis

Four data analysts, including the first author and three research assistants, independently reviewed the transcripts and conducted data analysis according to an iterative four-stage scheme developed by Biggerstaff and Thompson (46). Stage 1 involved the first encounter with the texts. We attempted to suspend presuppositions and judgments when reading the texts and focus on what was actually presented in the transcripts. We made notes of thoughts, observations, and reflection that occurred while engaging with the text. At Stage 2, we moved on to re-read the transcripts and identify preliminary themes that captured the essential features of the interviews. We also looked for possible connections between themes at this stage. Occasionally, contradicting narratives were identified in some participants, prompting us to revisit earlier transcripts in case something vital had been missed or misunderstood. At Stage 3, we grouped related themes together as clusters or concepts, which provided an overall structure indicating a hierarchical relationship between the themes. The final stage involved tabulating themes in a summary table with quotations that best captured the essence of the participants’ thoughts and feelings about their PBL coaching and learning experiences. The scheme was repeatedly applied to all the interview data until we were achieved a consolidated list of categories from the cyclical analysis.

### Ethical considerations

The research protocol was approved by the Research Ethics Committee of East China Normal University and Tongji University. All participants completed informed consent forms that included an introduction of the research purpose and explanations of the commitment involved in participation. All participants were reassured of the confidentiality and the right to withdraw at any time during the interviews. Each interviewee was given 50 RMB for their participation in the study.

### Reflexive statement

As Creswell stated, ‘one cannot escape the personal interpretations brought to the qualitative analysis’. Inevitably, we interpreted the interview transcripts through our own unique lenses (47, 48). We created the meaning of individual participant's experience by using the store of past knowledge that we brought to the new and interactive situation. For instance, how PBL approaches develop and how Chinese learners usually perceive medical education might have a great influence on how we situated and interpreted interviewees’ responses from a socio-cultural perspective. Moreover, we were cautious about the potential influence of our expectations on the effectiveness of coaching, which could affect the process of data collection and interpretation. By clarifying the nature of the study (exploratory rather than evaluative), we took a reflexive position in understanding what the participants actually said about their experiences rather than what we wanted to hear. After each interview, we wrote journals reflecting initial impressions and interaction with the participant. The notes evolved throughout the process, covering our reactions to the participants, our thoughts about themes that might be emerging, and our positions as interviewers. The reflective journals were maintained until the study was completed. After completion of the study, individual meetings with the participants confirmed that the findings accurately reflected their experiences.

## Results

The study results of IPA included a set of themes that have been developed after a series of coding. We identified six main themes with sub-themes regarding the medical students’ and tutors’ experiences of PBL coaching. The themes are presented in Table 1. To provide an insight into the themes, extracts were selected on the basis of being representative and interesting illustrations of each of the main themes.

**Table 1:** The themes in students’ and tutors’ experiences of PBL coaching

Super-ordinate theme	Sub-ordinate theme
1. Mindsets of coaching and learning	Positive attitude of coaching Resistance of change Mixture of lecturing and coaching
2. Development of learning dispositions and capacities	Lifelong learning dispositions Higher-order learning capacities Professional abilities in medical/clinical settings
3. Student group collaboration	Group dynamics Tutor facilitation in group work The special role of student hair
4. Coaching relationship	Multi-faceted relationship Mutual feedback
5. Personal and professional development	Psychological health and well-being Medical humanities and empathy Professional identity
6. Challenges and difficulties in implementation	

### Theme 1: mindsets of coaching and learning

Students and tutors commented on their general impressions of the PBL coaching process that reflected their basic mindsets of coaching and learning. Three sub-themes became apparent: perceived positive attitude of coaching, some resistance of change, and a preference of mixing lecturing and coaching.

The tutors showed a positive attitude toward PBL coaching, and most student participants seemed to embrace the idea of coaching. They agreed that instruction and guidance was essential, and that direct telling should be avoided:

This [coaching] method guided students to learn actively. We emphasized inspiring and illuminating questions, and we encourage students and positively guide them to think. This is what we lack in our educational system. When I was teaching the students following a traditional method in the ward, they seemed to be lost immediately. But this year, when I was coaching, I felt that the students were quite capable, completely different from the previous students. (T5)

However, there was a strong influence of traditional teaching and learning on their actual behaviors in the PBL coaching process. Some participants were familiar and comfortable with didactic lecturing and knowledge transmission. Most participants mentioned that it was difficult to change the conventional mindset immediately and there should be a transitional period. A mixture of coaching and traditional lecture-based learning seemed to be an ideal implementation in the current PBL environment. The point is to guide the students’ thinking process timely and encourage them to critically think about ‘clinical facts’ rather than merely transmit inert knowledge:

A senior tutor told us that talking too much or talking too little is equally inappropriate. My experience is that there should be some talking… I think we need to lead the thinking process. So I don't think that talking less is more. We need to talk to scaffold student thinking. Maybe I should do a bit of coaching and a bit of lecturing, trying to mix them in a way that fit the students’ needs. (T4)

### Theme 2: development of learning dispositions and capacities

The students and tutors expressed that they have developed a variety of learning dispositions and capacities throughout PBL coaching. These included a number of positive lifelong learning dispositions such as learning agency, self-directedness in learning, self-reliance and independence, and creativity.

When we faced a completely new case, we needed to imagine bravely what kind of disease it could be from available information. It inspired us to develop imagination and creativity… We analyzed the case little by little, and it was an exercise for our logical thinking and how to use the learned knowledge. (F4)

Most students mentioned that, through coaching, they developed specific intellectual and cognitive capacities such as logical thinking, critical thinking, effective use of current knowledge, and information management. These learning capacities might underlie a number of distinctive higher-order learning capacities. Moreover, the students reported the development of professional skills such as clinical reasoning ability and diagnostic ability. These included critical evaluation and selection of information resources and identifying the disease mechanism based on available information.

When we faced a completely strange case, and we hadn't learned it before, we might generate all kinds of hypotheses. Then the tutors would demonstrate a way of thinking, like how to search for evidence from symptom, examinations or other information relevant to the disease. It's like teaching us how to fish rather than giving us the fish. I felt that tutors not only supported us, they gave us a way of logical thinking, which was quite important. (F6)

The tutors resonated that information management was of critical importance to clinical criticality. Most of them thought that the provision of reliable resources for the students was mainly the tutors’ responsibility. However, one tutor disagreed and held a strong opinion that students should independently search for clinical information that is relevant to problem-solving. This reflected tutors’ different levels of cultivating self-directedness in students.

### Theme 3: student group collaboration

The students were encouraged to attend to the collaborative process through interdependent learning within the group. The theme of student group collaboration highlighted the complexity of students’ teamwork. Three sub-themes were identified: group dynamics, tutors’ group facilitation, and the special role of student chair.

Most participants enjoyed a sense of belongingness and developed a team spirit once their voices were heard, although the collaborative learning experience was not a smooth journey. The challenges were perceived as opportunities for developing interpersonal communication skills. Extroverts, such as outgoing, active students, were generally preferred in the groups for initiating discussions and reinvigorating the learning atmosphere by openly sharing their opinions. However, extroverts would seem to be more powerful and dominant in the group, and sometimes they overlooked the influence of their behaviors on others. An over-dominance of conversation and complex power relationships were perceived as problematic by some students. Although the students were all in their third year in medical school majoring in clinical medicine, they exhibited individual differences in gender, educational background before university, personality traits, and expertise. Students from different programs seemed to have difficulty in forming learning alliances, which might require more balanced dynamic and mutual understanding in the team.

Tutors’ coaching was found crucial in monitoring, managing, and regulating the group dynamics by providing observational information and necessary intervention. When students were not aware of their behaviors and attitudes and how they influenced the rest of the group, the tutors would intervene in the process. The participants agreed that tutors should take less control over the flow of group discussion and allow the students to work through the process themselves. Tutors should relinquish control but continue consistent observation and monitoring.

The tutor should be instructors of the field. The main discussion should be led by the students themselves, and then the students could arrange some activities around certain topics. If the discussion misses the point, or there is a big mistake in the direction, the tutors should correct the students. And the tutors should observe each student to help them or correct their mistakes. (M5)

In each group, there was a student chair, elected by the group members, who played a leading role in making overall arrangements of group work, organizing the information collected and questions generated by the members, allocating learning materials, and monitoring discussion process. The chair was elected again when the second case started. Being the student chair was reported to be a distinctive experience from being a group member. The chairs shouldered more responsibilities and took a more active role in collaborative learning. They demonstrated positive attitudes in terms of capacity development and knowledge building. Meanwhile, some students might feel vulnerable in taking up this role because being the chair meant being under more pressure, especially when the role was not self-selected. Moreover, being the chair might mean undertaking more information management and problem-solving tasks when other group members did not complete their share of work responsibly.

### Theme 4: coaching relationship

The participants noted that a satisfactory coaching relationship was established, maintained, and developed by the joint efforts of both students and tutors throughout the learning-facilitating process. The learning relationship between students and tutors was complicated and multi-faceted. At the beginning, it involved a clear apprenticeship as the tutors mentored the students on the principles of the course and offered more guidance. The relationship evolved toward a more equal relationship since students increasingly became adept and self-directed learners. Tutors were not necessarily playing a more authoritative role in the coaching relationship. The quality of the tutor–student relationship seemed to play an important role in the learning process. Students’ readiness and openness to coaching depended on their preferences about tutors. In addition, it was suggested that particular tutors should consistently work with one group of students so that the students became more familiar with each other and adaptive to different learning styles and habits throughout the course.

The tutor's role in this course was definitely different from the role in big lectures. In lectures, tutors are the main players. In this course, I think tutors were facilitators to students. Students were the learning agents. We just helped them, gave hints, and answered their questions when they didn't know. (T3)

The tutors were like coaches to some extent. They would coach you what you can do, but they wouldn't tell you directly how to do it. (M6)

The students and tutors were invited to provide feedback to each other in the last session, which was considered important for fostering the coaching relationship. The tutors sometimes gave feedback to students in the process of learning. A feedback form was designed by the administrative committee for students to provide their opinions on the overall quality of the course. However, the form was reported to be a disappointing tool. It only asked a few closed questions and listed evaluative scales. The problem of mutual feedback was constantly noted by students and tutors. It was suggested that there was a need for a more effective and authentic feedback.

### Theme 5: personal and professional development

This theme centered on the development of learners as whole persons, which involved students’ psychological well-being, education in medical humanities and empathy, and a continued cultivation of professional identities for both students and tutors. The tutors provided students with emotional scaffolding by understanding their personal learning styles, creating a harmonious and engaging learning atmosphere, and demonstrating warm regard toward individual students.

I felt supported in coaching… it was psychologically safe and open for me to discuss issues, not only the medical problems but also my own problems with understanding certain knowledge or with presentations in public. (F1)

The enhancement of psychological health and well-being was mostly achieved by tutors’ attentive listening, open questioning, motivational interviewing, use of humor, and other coaching skills. Interestingly, one tutor mentored the students with stress management skills and helped them to be psychologically resilient and better prepared for their medical career.

I sometimes tell [the students] my experience of coping with stress, like relaxation skills, thinking things in a brighter way… As you know, working as a doctor is a huge pressure. We are responsible for the patients, and we are responsible for many other people, and ourselves… We are very, very busy. When I am working in the ward, I cannot find time even for drinking water or going to toilet… Anyway, I tell the students what they will face when they become real doctors, and how to deal with the difficulties using my own experiences. I don't want them to be afraid… And coaching the students how to cope with stress is not something we usually do in PBL… But I just feel that I can do it in this course. (T4)

The majority of respondents noted that there was a stronger sense of medical humanities and empathy in PBL coaching. The tutors modeled interpersonal communication skills and demonstrated humanistic concerns in clinical practice, which greatly helped the students to develop an awareness of humanistic consideration, empathy, sensitivity to clinical issues, and a holistic view of patients.

In one case, the doctors went to the patient's home to observe his living environment. It might be one part of collecting the disease history… The tutors told us that doctors were not machines for treatment and that we need to communicate with patients and attend to their life. We should consider individual patient's condition more holistically. What we are facing is not a pile of physical examination results and papers, but a real patient, a human being. We should pay attention to him as a whole person. I felt humanistic consideration and empathy were really important throughout coaching. (F7)

The students reported that they were connected with an authentic sense of professional identity. It related to their deepened understanding of what kind of doctors they would become and what they could contribute to the clinical environment. Role playing was considered as a powerful coaching method of fostering professional identity.

Role playing was very useful. I had this idea spontaneously. You let the students take a real doctor's role and they became immediately engaged and had very strong motivation. They hope to be young doctors who can help their friends, families, and other people… And you encourage them to be real doctors in the future to treat diseases described in the cases and help patients to relieve the pain. They felt like really adopting their roles as doctors. Becoming good doctors takes generations after generations. I hope they would do a better job than us. (T4)

### Theme 6: challenges and difficulties of implementation

There were a number of barriers to implement PBL coaching in the current medical educational setting noted by the participants. The most frequent ones included limited time and resources, ambiguous criteria of assessment and insufficiently skilled facilitators. Most participants were concerned that PBL coaching might take up too much time in training and preparation. Another main challenge was that the assessment criteria were unclear for both student attainment and tutor effectiveness. For tutors, the evaluation of coaching effectiveness was ambiguous.

It seemed that there was no criteria of how we did in coaching, whether we were good coaches and whether we were coaching students in the right way. We need more professional guidance and evaluation in coaching. (T4)

The students were also not sure how they would be assessed. Most of them wanted to know the specific criteria so that they would know which areas needed self-improvement. However, one student claimed that knowing the criteria of assessment at the beginning of the course might endanger their intrinsic motivation in learning. Finally, the tutors recognized that the coaching was a potentially effective facilitation paradigm but their coaching skills seemed to be inadequate. Deciding when to intervene was perceived as a common difficulty. Sufficient training and experience in coaching were suggested in order to enhance the quality of intervention.

## Discussions

The current IPA study investigated the medical students’ and tutors’ experiences and views of the PBL coaching process. We identified six main themes that uncovered the multi-faceted nature of the PBL coaching process perceived by the participants. Themes 1 and 2, together, refer to a similar mindset shared by PBL coaching and PBL tutoring to support students’ self-directed learning. Themes 3 and 4 uncover the characteristics of coaching relationship between tutors and students, the learning dynamics among students themselves, and coaching skills needed to ensure the healthy functioning of PBL groups. Themes 5 and 6 address that coaching accentuates enhancing students’ emotional, psychological, and empathetic aspects in medical education, although it may face several practical challenges in implementation. The following discussion synthesizes the themes and attempts to address considerable similarities between PBL coaching and PBL tutoring as well as the particular contributions that coaching makes to the learning process.

### Coaching to support student self-directed and self-regulated learning

It is apparent in participants’ comments that PBL coaching, similar to PBL tutoring, aims to foster students’ capacity for self-directed and self-regulated learning. One of the distinguishing features and attractions of PBL in medical education is its potential to foster self-directed learning as a lifelong habit (3). In order for the student-centered approach to be realized, students must make the shift to their new roles as active learners and develop self-regulated learning skills (49). This notion is reflected in the theory of andragogy (31, 50) that adult learners are self-directed and goal-oriented. In PBL, students become responsible for their own learning, which necessitates reflective, critical thinking about what is being learned (51). Self-directed and self-regulated learning is commonly associated with intrinsic motivation, which occurs when students work on a task motivated by their own interests, challenges, and sense of satisfaction and when their needs for autonomy, competence, and relatedness are met (52). We found that students could perform extrinsically motivated actions with an attitude of willingness that reflected an inner acceptance of the value or utility of tasks. Thus, two kinds of motivation were also important for self-directed and self-regulated learning on two external regulatory levels: identified regulation and integrated regulation (53). When students consciously valued learning actions as having personal importance (e.g., engaging in collaborative discussion was important for a sense of belongingness to the team), they identified and internalized views of others in relation to the actions. If learning actions were congruent with students’ identity and synthesized with the self (e.g., the professional identity of being a good doctor), then the actions were regulated in an integrated way. These two kinds of actions are still viewed as extrinsically motivated because they are performed to achieve particular outcomes rather than because they are experienced as inherently interesting and enjoyable. However, we consider that these two types of motivation may be more pervasive than ‘purely’ intrinsic motivation in formal educational settings as students are required to achieve certain learning outcomes evaluated by various forms of assessment, whether formative or summative. As Evensen proposed, self-directed learning may be an individual characteristic that changes over time, for better or worse (54). Therefore, the point here is coaching to foster identified and integrated motivations, and internalize a sense of both responsibility and the value of extrinsic goals, as well as intrinsic motivation, in order to catalyze students’ self-directedness and self-determination in learning. Tutors can provide intentional support by consciously cultivating behaviors, goals, beliefs, and strategies that lead to self-directed and self-regulated learning and establishing a dynamic, reciprocal coaching relationship with students.

### The coaching relationship between tutors and learners

The findings showed that the dynamics of the coaching relationship differed from the overtly hierarchical relationship frequently associated with teaching or lecturing in the clinical settings. The coaching process was created moment-by-moment as tutors and students worked together through a supportive means and style of communication (55). There was a gradual transition toward the coaching relationship as noted in the study. At the beginning, the tutors took a leading role in launching the problem and offered more direct guidance, then provided ongoing monitoring and observation of guided collaborative inquiries and solution creation in the student groups. Finally, they carefully reduced the amount of direction they provided as the students became more experienced learners. In this aspect, PBL coaching may not be significantly different from PBL tutoring, in which tutors also provide students with appropriate structure, scaffolding, and guidance, so that students simultaneously begin to develop self-directed and self-regulated learning skills and knowledge construction through problem-solving. PBL coaching might differ from PBL tutoring in that coaching expressly stresses the quality of coaching relationship between students and tutors, interpersonal communication ability, and the personal qualities of coaches (40). The students indicated that how they perceived the quality of the relationships with their tutors could determine whether they felt sufficiently open to the challenges in PBL. We found that tutors’ interpersonal communication ability was a critical element in managing group dynamics and modeling effective interactions for students, which was contrary to Van Berkel and Dolmans’ study that tutors’ interpersonal behavior and stimulation of contextual learning did not significantly contribute to better group functioning (56). Tutors’ personalities, friendliness, approachability, and social congruence were also found to be essential in forming a learning partnership in PBL coaching. This is related to the notion of genuineness, congruence, or authenticity from a humanistic perspective of coaching (19). Nonetheless, the limited ability of the tutors, steeped in the traditionalist epistemology, in fully understanding the change in role from lecturers to coaches could create an authority-dependency situation (57). This issue was confirmed by the over-reliance of some student participants on direct guidance. The extent to which tutors could make the role transition was found to be a major factor in successful PBL coaching. PBL coaching requires adequate students’ and tutors’ preparedness with a balance of learner- and tutor-directed learning. Not only should tutors be trained to put on a ‘coach's hat’, but students should also be suitably induced into the PBL learning process (58, 59) and told what they should anticipate from the coaching experiences.

### Facilitative skills used in PBL coaching

To our surprise, the participants did not explicitly comment on coaching skills and strategies. It might be because they have received limited training of coaching and they were not aware of the importance of exercising various coaching skills and strategies in PBL. PBL coaching requires a number of basic facilitative skills as required in PBL tutoring, including scaffolding knowledge construction, supporting metacognitive development, and open-ended and reflective questioning designed to help students make their thinking visible, modulating the challenge of the learning to meet students’ requirements, and monitoring students’ progress and group dynamics (9). The tutors could mainly take a wandering facilitation mode (60). They rotate from group to group, adjusting the time spent with each group in the classroom according to students’ needs, and dynamically assessing the progress of each group and readjusting their facilitation efforts accordingly. Similar to PBL tutoring, PBL coaching also requires the facilitators to ensure that all students are involved in the discussion and exercise distributed expertise (61, 62). As the students divide up the learning issues, they are able to learn to become ‘experts’ in particular topics and bring the results together, so that the whole group could tackle problems that are normally considered too difficult for each student alone. The difference lies in that in PBL tutoring, expert information, and guidance can be offered directly to the learners through conversations or explanations of the rationale underlying the reasoning processes (3), whereas PBL coaching greatly emphasizes indirect facilitation and passing the leading role to the student chairs, in each group. The student chairs develop their facilitative skills throughout the course because they function as ‘junior tutors’. After consulting with the tutors, the chairs assign group members to clearly different roles, such as forming problems, summarizing results, and relating predictions to theories, in order to ensure that all group members are cognitively engaged rather than merely talking together.

### Addressing emotional, psychological, and empathetic aspects in medical education

What coaching contributes to PBL is that coaching accentuates that learners’ cognitive, affective, psychological, relational, and social needs should be appropriately addressed and supported with appreciation, facilitation, and positive regard in their own learning contexts. According to many PBL instructional documents in a variety of contexts, PBL has emphasized the importance of understanding content, disciplinary epistemologies and investigative strategies, the nature of scientific research, and the practices involved (1, 3). PBL tutoring, therefore, is primarily designed to fulfill these learning goals, and it is centered on facilitating the cognitive and motivational processes in student learning. However, the existing literature on PBL has largely neglected the emotional and psychological aspects (63). Strong psychological and interpersonal support is especially needed within the particular context of the Chinese medical working environment. Medical workers including clinicians, nurses, social workers, and other staff in the hospital generally withstand enormous pressure and bear very intense schedules. They also need to deal with various emergencies and face urgent problem-solving situations during the day. The presence of burnout, depression, and stress has been shown to relate to fundamental attributes of medical professionalism (64, 65). Therefore, the promotion of personal psychological health and well-being is crucial for their healthy and optimal functioning. PBL coaching explicitly involves promoting medical humanities, cultivating empathetic dispositions, enhancing psychological health and well-being, and fostering a strong career identity with higher self-respect. This study provided empirical evidence that PBL tutors could be personal coaches to individual students, embracing their distinctive emotional, psychological, and social needs in addition to the needs that are usually involved in PBL tutoring. The coaching was conducted in a well-structured learning environment, and through empathetic listening skills and positive regard toward individual students, mentoring stress management skills, and role-modeling in experiential learning.

Empathy is increasingly understood to be fundamental to effective medical education and the foundation of patient–doctor relationship (66, 67). Empathy is considered an innate ability that can be cultivated, practiced, and perfected in coaching, counseling, and psychotherapy literature (18, 19, 24, 36). Although PBL tutoring involves communication skills training to some extent, it has been mainly regarded as a way to provide better cognitive scaffolding, rather than an essential ingredient to develop and sustain therapeutic doctor–patient relationships (68) and an interdependent quality of a competent clinical practitioner (69). In this study, the tutors undertook empathetic positions in tutor-student interactions and became role models in approaching clinical situatons with compassionate reasoning. PBL coaching is potentially a powerful pedagogical approach for students to experience, observe, imagine, and reflect on medical empathy. They would assimilate the empathetic skills into their repertoire and then transfer these skills into other contexts of learning and future clinical practice.

In addition, it seems that there was more tolerance of errors in PBL coaching. Chi et al. suggested that errors are a necessary step in learning to apply new knowledge (70). In traditional Chinese education, making mistakes in the classroom is regarded as embarrassing and humiliating for both students and tutors. Chinese PBL tutors may still try to guide students to find the right solutions and discreetly avoid making mistakes themselves. In contrast, PBL coaching creates a more flexible, secure, and friendly learning environment in which students and tutors are allowed to acknowledge that they do not know all the answers, embrace mistakes that they make, and clarify any interpersonal misunderstanding. By articulating incorrect knowledge and sharing the lessons learned from these mistakes, students have the opportunity to revise their false beliefs, foster correct knowledge, and play with new ideas. Moreover, by openly recognizing their vulnerability, students and tutors could deepen the trust and rapport between themselves, which in turn helps to create an empathetic and nurturing atmosphere of learning.

In summary, we propose that medical educators and faculty should pay more attention to identifying the possible cognitive, motivational, emotional, and psychological responses of the individual medical student as a whole person and how these responses may inform learning experiences, thus resulting in changed learning behaviors and learning outcomes.

### Limitations of the study

There are two primary limitations of the current study. First, the research findings may not be generalizable, given the nature of IPA methodology. However, the study may shed light on the implementation of innovative approaches in PBL facilitation based on the personal experiences of medical students and tutors. It may advance our understanding of how we could address the challenges and improve PBL facilitative processes in the real educational context from different angles, particularly from the perspective of coaching psychology. The second limitation lies in the comparison between PBL coaching and tutoring approaches. We did not employ a controlled experimental or a quasi-experimental design by simultaneously conducting PBL coaching and another alternative PBL facilitation method in two independent populations. A more rigorous experimental design is perhaps needed in order to elucidate the distinction between PBL coaching and tutoring in the medical education context.

### Implications and future research

The results of this study have implications on three levels. For PBL tutor recruitment, training, and continuous professional development, it seems to be desirable to include and strengthen coaching psychology such as person-centered, cognitive behavioral, and narrative approaches. From a PBL facilitation perspective, tutors need to intentionally and flexibly incorporate a range of coaching skills, strategies, and models into their practices intended to support students in a holistic way. On the level of educational administration, it is important to provide sufficient learning resources and financial support, train more qualified tutors, and allow adequate time for completion.

A future research agenda of PBL coaching includes a more careful examination of tutors’ facilitation and how the elements of coaching can be tailored to different goals, local contexts, and developmental levels of learners. There is a great need for evidence-based instructional strategies with which medical educators could make informed choices in adapting coaching to their particular PBL approaches.

## Conclusions

This paper has focused on medical students’ and tutors’ experiences and perspectives of PBL coaching in the context of undergraduate medical education via IPA methodology. The results, through a number of main themes, have revealed that PBL coaching, as a systematic facilitative method, offers opportunities for students and tutors to become reflective co-learners and flexible thinkers. PBL coaching empowers medical students to consider how they would use constructed knowledge and specific learning dispositions in real clinical cases through using self-directed and collaborative learning skills. The results also showed that, although PBL coaching has a significant overlap with PBL tutoring, it has the advantage of suggesting a method to address medical humanities and empathy, attend to effective communication in clinical environment, enhance psychological well-being, and foster professional identities of medical students in a more sensitive, comprehensive, and professional way.
